# Comparative analysis of ampoules and vials in sterile and conventional packaging as to microbial load and sterility test

**DOI:** 10.1590/S1679-45082016AO3484

**Published:** 2016

**Authors:** Raphael Ribeiro de Aquino Freitas, Maria Angela Tardelli

**Affiliations:** 1 Universidade Federal de São Paulo, São Paulo, SP, Brazil.

**Keywords:** Infection control, Anesthesia, Contamination, Anesthesia, conduction

## Abstract

**Objective:**

To compare sterility and microbial (bacteria and fungi) load in the outer part of hyperbaric bupivacaine (Neocaína^®^) in ampoule and bupivacaine in vial, in conventional and sterile pack formulations.

**Methods:**

The sterile packs were divided into two groups: G1 (n=16) with ampoules and G2 (n=16) with vials. Conventional formulations were divided into two groups, being G3 (n=16) with ampoules and G4 (n=16) with vials. The ampoules and vials were opened and had their content drawn. The empty bottles were then placed in sterile plastic bags and sent for analysis of microbial load (bacteria and fungi) and sterility testing. Data were analyzed using the χ^2^ test with Yates correction, and 95% confidence interval.

**Results:**

G1 and G2 showed no bacterial growth when compared to conventional groups (p<0.001). The most common agent in conventional microbiological samples was *Staphylococcus aureus*. There was no fungal growth in both groups.

**Conclusion:**

The use of (sterile pack) reduces the microbial load of bottles, and would decrease the chance of exposure to potential contamination of the anesthetic solution.

## RESUMO

**Objetivo:**

Comparar a esterilidade e a carga microbiana (bactérias e fungos) da parte externa dos frascos de envasamento de bupivacaína hiperbárica (Neocaína^®^) em ampola e bupivacaína em frasco-ampola das apresentações convencional e estéril (*sterile pack*).

**Métodos:**

As apresentações estéreis (*sterile pack*) foram distribuídas em dois grupos, sendo que o G1 (n=16) continha as ampolas e o G2 (n=16), os frascos-ampola. As apresentações convencionais foram distribuídas em dois grupos, a saber G3 (n=16) com as ampolas e G4 (n=16) com os frascos-ampola. As ampolas e os frascos-ampolas eram abertos e tinham seu conteúdo aspirado. Os frascos vazios eram, então, acondicionados em sacos plásticos estéreis e enviados para análise quanto à carga microbiana (bactérias e fungos), bem como para o teste de esterilidade. Os dados foram analisados por meio do teste χ2 com correção Yates com intervalo de confiança de 95%.

**Resultados:**

Os grupos G1 e G2 não apresentaram crescimento bacteriano quando comparado aos grupos convencionais (p<0,001). O microbiano mais comum nas amostras convencionais foi o *Staphylococcus aureus*. Não houve crescimento de fungos em nenhum dos grupos.

**Conclusão:**

O uso de embalagens estéreis (*sterile pack*) diminui a carga microbiana dos frascos de envasamentos, o que diminuiria a chance de exposição a uma potencial contaminação da solução anestésica.

## INTRODUCTION

In the past years, modern medicine has used spinal (peridural, subarachnoid or dual block) anesthesia in many situations. It is used primarily in obstetrics, gynecology and lower limb surgeries, as well as in treatment of acute and chronic postoperative pain.^(^ ^1^ ^)^ However this technique might present complications, including some severe events, such as traumatic nerve lesions, peridural hematomas, infections like peridural and paravertebral abscess, and acute bacterial meningitis.^(^ ^2^ ^-^ ^5^ ^)^

Endogenous or exogenous sources of microorganisms may enter the subarachnoid or peridural spaces by direct inoculation, hematogenous dissemination from other sites or migration through the catheter, via skin or tissue subcutaneous. Several case reports suggested the microorganisms of the patient’s or anesthesiologist’s microbiota can be directly inoculated when the needle or the catheter is inserted in these spaces, or when the anesthetic vial solutions are administered to patients, and the outer part of vials is not sterile.^(^ ^6^ ^)^ Many investigators collected cultures of needles, syringes and tubes used to administer regional anesthesia, aiming to check when these items become contaminated for use and, consequently, may be a source of infection. Some studies demonstrated that the incidence of device contamination ranged from zero to 33%, but no investigator identified infected patients. In addition, they could not correlate the source of contamination with infection.^(^ ^6^ ^,^ ^7^ ^)^

There are reports in the literature of cases of meningitis after regional anesthesia.^(^ ^2^ ^)^ There are multiple mechanisms proposed as source of meningeal infection. First, the microorganism may be introduced during the insertion of a contaminated needle or catheter, which could explain most cases related to spinal anesthesia. The microorganisms involved are *Staphylococcus aureus* and *Streptococcus* spp., described in more than 50% of cases. Sometimes the origin of these microorganisms is the physician’s nasopharynx.^(^ ^8^ ^)^ This situation may occur when asepsis measures during the procedure are not adequate, such as the team involved in block aesthesia does not wear the mask correctly. Second, needles and catheters can be contaminated by bacteria that live on the skin and may later migrate along the skin surface to the subarachnoid space.^(^ ^9^ ^)^ This would explain most infections secondary to chronic spinal analgesia, and the most common agent is *Staphylococcus aureus*. Third, there may be a hematogenous dissemination of a distant source of infection, and contamination of the subarachnoid space occurs with blood flow during puncture.^(^ ^10^ ^)^ Finally, the infusion of contaminated substances was the cause in a few cases, and some of them were fatal.^(^ ^10^ ^)^ The etiologic spectrum of meningitis associated to regional anesthesia is broad, including viridans group *Streptococcus*, other species of *Streptococcus*, *Staphylococcus aureus*, *Pseudomonas* spp., *Enterococcus faecalis*, *Corynebacterium*, *Acinetobacter* and even *Aspergillus*.^(^ ^11^ ^)^

Although rare, the infectious complications of regional anesthesia can be devastating. The anesthesiologists play an important role in prevention of nosocomial infections. In the anesthetic practice, invasive procedures - such as tracheal intubation, venous access or blocking nerve bundles - are routinely performed and they break through physiologic barriers, allowing contamination of the patient by microorganisms and development of infection. Non-compliance with the recommended practices may facilitate transmission of microorganisms from the anesthesiologist to the patient, from the patient to the anesthesiologist, and among patients.^(^ ^12^ ^)^ Hygiene practices by professionals, proper cleaning of equipment and appropriate performance of invasive procedures are relevant aspects to reduce the risk of transmitting infection.^(^ ^6^ ^)^

The local anesthetics and opioids that are usually administered by peridural or subarachnoid routes are available in recipients, whose external parts are exposed to environmental pathogens, and are a potential source of contamination. Opening and handling the ampoules are generally not performed in a standardized manner. Possible contamination can occur during the several stages of the process, involving handling of the ampoule up to administration of its content.

## OBJECTIVE

To compare sterility and microbial (bacteria and fungi) load in the outer part of hyperbaric bupivacaine (Neocaína^®^) in ampoule and bupivacaine in vial, in conventional and sterile pack formulations.

## METHODS

The study was carried out in the operating room of *Hospital São Paulo*, under coordination of the anesthesia service, Department of Anesthesiology, Pain and Intensive Medicine, *Universidade Federal de São Paulo*, *Escola Paulista de Medicina*, under protocol number 0860/11, approved by the Research Ethics Committee.

The sterile packs of Neocaína^®^ (C*ristália Produtos Químicos e Farmacêuticos*, São Paulo, Brazil) were distributed into two groups: G1 with 4mL ampoules of 0.5% bupivacaine hydrochloride + 8% glucose) and G2 with 20mL vials of 0.5% bupivacaine hydrochloride without vasoconstrictors).

The conventional formulations of Neocaína^®^ (*Cristália Produtos Químicos e Farmacêuticos*, São Paulo, Brazil) were distributed into two groups: G3 with ampoules and G4 with vials.

The analysis was carried with 16 samples of each group, totaling up 64 samples. The samples were collected between 7:00 a.m. and 11:00 a.m., during 5 days. These drugs were delivered from the pharmacy of the *Hospital São Paulo*, through the natural flow of medications and were not necessarily from the same batch.

The samples were carefully identified considering date and time, kept under refrigeration and sent to the laboratory.

**Figure 1:**
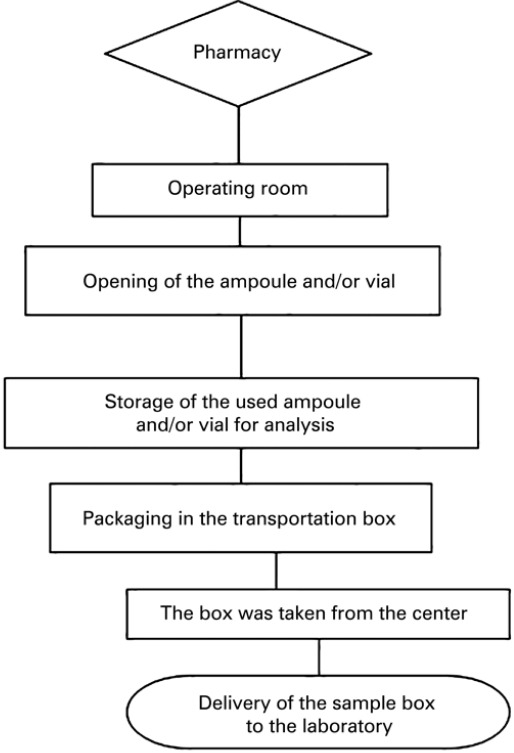
Sample flow

The sample flow is displayed in figure 1.

The anesthesiologist wore cap and mask for collection and did hand asepsis with chlorhexidine before handling the medication, according to the routine of the operating room, and put on sterile gloves. One assistant, appointed by the anesthesiologist, removed and handled the conventional ampoule and vial. The principal investigator assured standardization of collections in the different groups.

In groups G1 and G2, the assistant opened the sterile pack of the ampoule and vial, and placed them on a sterile tray. The anesthesiologist broke the ampoule neck to withdraw all content using a 5mL syringe, or removed the vial cap to withdraw the whole content using a 20mL syringe. After this procedure, the anesthesiologist placed the sterile pack ampoules and vials inside sterile bags.

In the conventional groups G3 and G4, the recipients were not cleaned before use, according to usual clinical practice. In the group G3, the assistant opened the ampoule and held it for the anesthesiologist to withdraw the content using a 5mL syringe. In the group G4, the assistant removed the cap of the vial and held it for the anesthesiologist to withdraw the content using a 20mL syringe. Then the assistant placed the conventional ampoules and vials in sterile bags.

After closure, the sterile bags were stored in a refrigerator at +4°C-+8°C, until all samples of the day were collected. Later they were sent to the laboratory *Controlbio Assessoria Técnica Microbiológica S/S Ltda*., which complies with the international standards ISO 11137-1:2006 and ISO 11137-2:2006.

Although the initial sample collection was not blinded due to the handling routine, the laboratory staff that analyzed the samples was not aware of the groups.

Qualitative and quantitative analyses of ampoules and vials were performed for microbial load on their outer surface. Under rotation, the vials were placed on blood agar plates, incubated at 30°C -35°C, for 72 hours. Afterwards the plates were read in a colony counter.

The microorganisms were identified by biochemical tests and culturing for fungus, and BBL Crystal™ for bacteria.

For the sterility test, ampoules and vials were immersed in 50mL of a liquid medium (Soybean-Casein Digest Medium or similar). These samples were incubated at 30°C -35°C, for 14 days. After this period, the culture medium was assessed regarding turbidity. An aliquot was withdrawn from the turbid vials for subculture and identification of microorganisms, by means of Gram staining and BBL Crystal™ kit.

Date were analyzed using the Yates corrected χ^2^ test and 95% confidence interval.

## RESULTS

The results were analyzed according to the study groups.

### Sterile pack *versus* conventional ampoule

Bacterial growth was observed in 13 out of 16 (81.25%) conventional ampoule samples; in that, seven were *Staphylococcus aureus* seven *Bacillus* spp and one had *Micrococcus* spp. Some samples had different species. No fungal growth was reported in the samples.

The sterile pack ampoules had no bacterial or fungal growth in the assays. This difference in the χ^2^ test was 18.656, with one degree of freedom and p<0.0001.

### Sterile pack *versus* conventional vial

Fifteen out of 16 (93.75%) conventional vial samples had bacterial growth. Twelve samples with *Staphylococcus aureus* six with *Bacillus* spp and three with *Micrococcus* spp, and some samples had different species. No fungal growth was described in the samples.

The sterile pack vials had no bacterial or fungal growth in the assays. This difference in the χ^2^ test was 24.596, with one degree of freedom and p<0.0001. Table 1 shows the microbial culture results in the conventional groups.

**Table 1:** Distribution of microorganisms identified in the conventional groups

	*Staphylococcus aureus*	*Bacillus* spp	*Micrococcus* spp
	n (%)	n (%)	n (%)
Ampoule (n=16)	7 (54)	7 (54)	1 (6)
Vial (n=16)	12 (80)	6 (40)	3 (20)

## DISCUSSION

The introduction of pathogens in the neuroaxis may occur by three different ways: skin contamination and subsequent dissemination through the needle or catheter; direct extension or hematogenous dissemination of distant foci; or by injection of a contaminated solution.^(^ ^13^ ^)^ The latter is the least frequent cause^(^ ^14^ ^)^ and has been scarcely reported in the literature.

Hence this study aimed to compare the microbial load of vials in sterile or conventional packaging, and assess the vial contribution to exposing liquids to pathogens during their handling.

Sterile pack is the name of anesthetics and adjuvants packages submitted to sterilization before their use in clinical practice. It is a chemical sterilization process, at low temperature, using hydrogen peroxide. The free radicals generated from hydrogen peroxide interact with molecules that are essential for the metabolism and reproduction of microorganisms. They make unspecific chemical bindings with cytoplasm membranes, enzymes, deoxyribonucleic acid (DNA), ribonucleic acid (RNA), among others, resulting in sporicidal, fungicidal, bactericidal and virucidal actions. It is a feasible and quick sterilization process.

As to microbial load, the results showed no pathogen growth in sterile pack ampoules and vials when comparing to conventional recipients. This finding would contribute to lower risk of microbial contamination in anesthetic solutions. However, this article has a limiting factor: it was not possible to confirm the relevance of solution contamination dependent on the type of package, because the methods used in the present study did not include analysis of the ampoule and vial contents.

A previous study had already demonstrated the likelihood of contamination of the injected solution due to a contaminated ampoule is approximately 1.66%. This would require a 15-fold larger sample in our study.^(^ ^15^ ^)^ Further studies must be conducted to prove this causal relation.

Although rare,^(^ ^16^ ^)^ complications of microbial infections during anesthesia must be considered, and meningitis is one of the most important. The literature describes *Staphylococcus aureus* as the most frequent etiology of complications during neuroaxis regional block.^(^ ^9^ ^,^ ^17^ ^)^ This datum corroborates the findings of the current study, since this agent was found in 54% of conventional ampoule and in 80% of conventional vial samples. There was no growth of *Streptococcus* spp probably due to the team wearing facial masks when handling vials during the anesthetic blocks, as per the protocol.

Although infection during neuroaxis anesthesia may occur during insertion of the needle, or because of failures in sterile techniques, there are reports on injection of contaminated solution.^(^ ^18^ ^)^ This fact is not broadly disseminated, but such injection may lead to devastating infectious complications in regional anesthesia. When handling all materials, the anesthesiologists should give priority to practices that minimize contamination.

## CONCLUSION

The use of sterile packages can reduce the exposure to potential contamination of infused anesthetic solutions. Besides that, anesthesiologists will demand less assistance and, consequently, will feel more confident during the preparation of material needed for regional anesthesia.
